# Longitudinal observation of viral load in patients infected with Omicron variant and its relationship with clinical symptoms

**DOI:** 10.3389/fmicb.2022.1037733

**Published:** 2023-01-13

**Authors:** Kai Zhou, Bingjie Hu, Xinzhuan Zhao, Hongbo Chi, Juan Pan, Yufen Zheng, Xiaojie Bi, Mengyuan Chen, Jicheng Xie, Jiaqin Xu, Tao-Hsin Tung, Bo Shen, Hongguo Zhu

**Affiliations:** ^1^Taizhou Hospital of Zhejiang Province Affiliated to Wenzhou Medical University, Linhai, Zhejiang, China; ^2^Evidence-based Medicine Center, Taizhou Hospital of Zhejiang Province Affiliated to Wenzhou Medical University, Linhai, Zhejiang, China

**Keywords:** Omicron variant, nucleic acid Ct value, time to peak viral load, duration of this Omicron variant, asymptomatic infected patients

## Abstract

**Objective:**

In 2022, a new coronavirus variant (Omicron) infection epidemic broke out in Shanghai, China. However, it is unclear whether the duration of this omicron variant is different from that of the prototype strain.

**Methods:**

We retrospectively analyzed 157 cases of Omicron variant infection in Taizhou Public Health Center from March 29, 2022, to April 18, 2022, and observed the dynamics of nucleic acid Ct values during the admission and discharge of patients. Clinical and laboratory indicators of these patients were also obtained.

**Results:**

Compared to the prototype strain, the Omicron variant showed a broad population susceptibility in infected individuals (regardless of age and presence of underlying disease) and had slight damage to the immune system and renal function; the viral loads peaked was 2-3 days from disease onset; the median duration of omicron variant was 15-18 days; the nucleic acid Ct value of nasopharyngeal swabs of infected patients is lower than that of throat swabs, and the Ct value of oropharyngeal swabs is unstable during the recovery period.

**Conclusion:**

Therefore, we found that the time to peak viral load of this Omicron variant was 2-3 days after the onset of the disease, and the duration was 15-18 days; symptomatic patients had higher viral load and longer hospitalization time. This finding will provide a basis for understanding omicron variants and formulating the national prevention and control strategy.

## Introduction

The first case of SARS-Cov-2 infection emerged in December 2019 and became a global public health event in 2020 following Severe Acute Respiratory Syndrome pneumonia (Mahase, 2020). In addition, new variants of the virus continue to emerge, with the currently predominantly prevalent SARS-CoV-2 Omicron variant first identified in South Africa, which has a more mutated genome than the wild type, accelerated transmission (Fan et al., 2022) and increased infectivity (Luo et al., 2021). There were 57,438 confirmed cases and 580 cumulative deaths in Shanghai, where a wave of infections started mid-to-late February (Shanghai Municipal Health Commission News release, 2022).

Studies have reported that Omicron variants have higher viral loads (Luo et al., 2021). Omicron’s exponential growth and increased infectivity are mainly attributed to immune escape due to altered spike-in antigens (Carreño et al., 2022; Dejnirattisai et al., 2022). However, increased viral load has also been found to contribute to the increased infectivity of previously emerged variants and to be associated with symptomatic versus asymptomatic infections (Teyssou et al., 2021). By assessing viral load based on reverse transcriptase PCR (RT-PCR) cycling threshold (Ct), Salvatore et al. found that prototype strain infections had lower Ct values in symptomatic individuals compared to asymptomatic ones and were negatively correlated with duration of illness (Salvatore et al., 2021). Luo et al. (2021) compared Ct values in respiratory specimens between the Alpha, B.1.2, and Delta variants. They found that Delta Ct values were lower than those of the Alpha variant (20.08 vs. 21.74, *p* < 0.05) but not statistically different from those of the B.1.2 variant.

The omicron cohort in this study was homozygous for the Shanghai COVID-19 patients infected in the same period. Phylogenetic features of SARS-CoV-2 viral genomes in this period, and inferring their relationship with those available on the GISAID database, indicated that all of the new viral genomes in Shanghai were clustered into the SARS-CoV-2 BA.2.2 sub-lineage (Zhang et al., 2022). There are few longitudinal studies on the viral load after Omicron infection and its relationship with clinical symptoms. The viral load correlates with the presence or absence of symptoms and has a specific impact on the course of the disease and patients’ infectiousness. Therefore, this paper further discusses the above aspects to guide clinical practice.

## Materials and methods

### Study cohort

**Figure 1:**
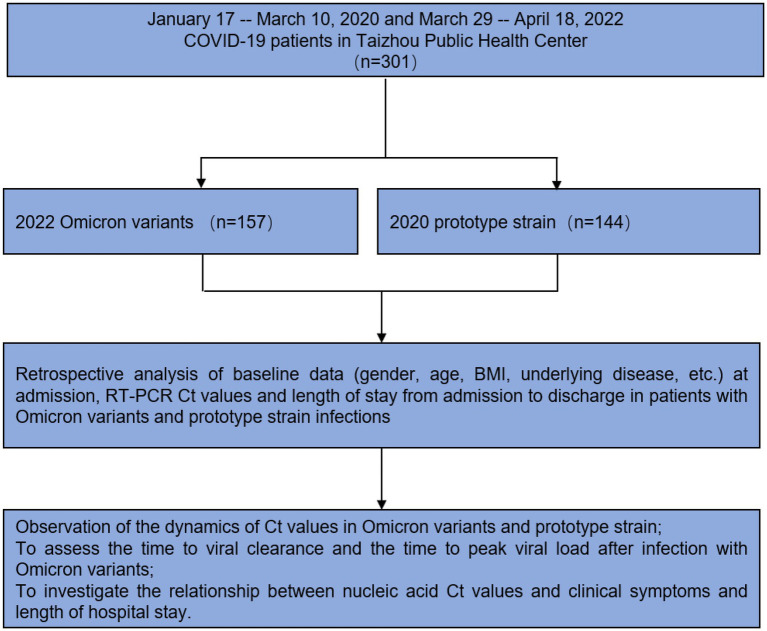
Study design and workflow.

A retrospective analysis was made on 301 cases of patients with COVID-19 who were admitted to Taizhou Public Health Center from January 17, 2020, to March 10, 2020, and from March 30, 2022, to April 18, 2022. The infected SAS-cov-2 virus strain divided the patients into 157 cases infected with the Omicron variant and 144 cases infected with the wild-type strain (prototype strain) (Figure 1).

**Table 1:** Demographic and clinical characteristics of the patients.

Characteristic	Omicron (*n* = 157)	Prototype strain (*n* = 144)	*p value*
Gender – ^a^ no. (%)			
Male	81 (51.6)	77 (53.5)	0.744
Female	76 (48.4)	67 (46.5)	
Median Age(IQR) – yr. ^b^	35.0 (28.0–39.0)	47.0 (38.0–56.0)	**<0.001**
Median BMI(IQR), kg/m^2^	22.5 (20.7–25.2)	24.4 (22.0–26.4)	**0.015**
Median Onset to admission days(IQR)	2 (2–3)	6 (4–10)	**<0.001**
Median length of stay(IQR), days	15 (13–18)	22 (13–28)	**<0.001**
Median length of stay of symptomatic	15 (13–19)	NA	
Median length of stay of asymptomatic	13 (12–15)	NA	
Symptoms and signs – ^a^ no. (%)			
Fever	71 (45.2)	114 (79.2)	**<0.001**
Cough	78 (49.7)	65 (45.1)	0.430
Pharyngalgia	66 (42.0)	17 (11.8)	**<0.001**
Headache	39 (24.8)	16 (11.1)	**0.002**
Expectoration	36 (22.9)	26 (18.1)	0.296
Stuffiness	30 (19.7)	7 (4.9)	**<0.001**
Muscle soreness	12 (7.6)	6 (4.2)	0.204
Chest tightness	4 (6.2)	11 (7.6)	**0.043**
Diarrhea	0 (0)	6 (4.2)	**0.010**
Heart rate	82 (75–86)	82 (75–91)	0.365
Respiratory rate	18 (18–18)	18 (18–20)	**<0.001**
Comorbidities – ^a^ no. (%)			
Hypertension	8 (6.3)	22 (15.3)	**0.003**
Diabetes	3 (1.9)	14 (9.7)	**0.003**
Cardiovascular disease	0 (0)	3 (2.1)	0.108
Cerebrovascular disease	0 (0)	3 (2.1)	0.108
Chronic bronchitis	0 (0)	4 (2.8)	0.051
Tuberculosis	0 (0)	4 (2. 8)	0.051
Malignant tumor	0 (0)	2 (1.4)	0.228
Thyroid disease	1 (0.6)	5 (3.5)	0.107
Hepatitis	0 (0)	7 (4.9)	**0.005**
Chronic kidney disease	0 (0)	2 (1.4)	0.228
Digestive system disease	2 (1.3)	7 (4.9)	0.092
Chest CT – ^a^ no. (%)			
Involvement of chest radiographs	64 (40.8)	142 (98.6)	**<0.001**
Image manifestations of COVID-19	15 (23.4)	142 (100)	**<0.001**
Pulmonary plaques	12 (18.8)	76 (53.5)	**<0.001**
Ground-glass opacity (GGO)	13 (20.3)	49 (34.5)	**0.046**
Pulmonary fibrosis	7 (10.9)	8 (5.6)	0.486
Pulmonary consolidation	0 (0)	32 (22.5)	**<0.001**
Laboratory data Median(IQR)			
Lymphocyte count	1.40 (1.13–1.90)	1.18 (0.80–1.58)	**0.001**
Red blood cell count	4.77 (4.45–5.17)	4.50 (4.18–4.96)	**0.001**
D-dimer	0.22 (0.16–0.26)	0.24 (0.18–0.41)	**0.005**
eGFR	117.0 (106.0–122.0)	95.0 (83.0–105.0)	**<0.001**

^a^no. (%): number. ^b^yr.: year. IQR, interquartile range. The meaning of the bold values is *p* ≤0.05.

### Data collection

Data on epidemiological, clinical, and laboratory characteristics were obtained through data collection forms in the hospital’s electronic medical records and reviewed by a team of trained physicians. Clinical information included personal characteristics (gender, age, and BMI), comorbidities, symptom onset date, and length of hospital stay. Underlying conditions had hypertension, diabetes, cardiovascular disease, lung disease, kidney disease, liver disease, and a history of prior malignancy. We considered that the first symptoms began with a fever, cough, sore throat, headache, sputum production, nasal congestion, muscle aches, and diarrhea. Laboratory characteristic data included complete blood count, blood gases, electrolytes, coagulation indicators, and organ function characteristics. The chest CT scan was performed using a combined 40-row UCT530 scanner (United imaging, Shanghai, China). The acquired CT images were sent to the GE-PACS workstation and read by two senior physicians in a double-blind manner.

### COVID-19 RNA analysis

The nucleic acid test Ct values of patients infected with Omicron variants or prototype strains were collected from throat swabs, nasopharyngeal swabs, and sputum and fecal specimens from admission to discharge.

RNA assay of the prototype strain was performed with throat swabs, nasopharyngeal swabs, sputum, and stool as test specimens. The sample nucleic acids were extracted by nucleic acid extraction reagent (Zhijiang Biological, Shanghai, China). The target gene was detected with the novel coronavirus 2019-nCoV nucleic acid detection kit (fluorescence PCR method) (Reagent No: SFDA: 20203400057, Zhijiang Bio, Shanghai, China). This kit uses RT-PCR (one-step method for reversing transcription-polymerase chain reaction) combined with Taqman technology to detect the novel coronavirus’s RdRP gene, N gene, and E gene. If the RdRp gene is positive (Ct ≤ 43) with E or/and N gene also being positive (Ct ≤ 43), the result is determined as positive.

RNA detection of the Omicron variants was performed mostly with throat and nasopharyngeal swabs as specimens. Nucleic acid was extracted by the novel coronavirus 2019-nCoV nucleic acid extraction kit (magnetic bead method) (Mingde, Wuhan, China) detection kit (fluorescence PCR) (Reagent No: SFDA: 20203400212, Wuhan Mingde Bio, Wuhan, China) for testing. This kit uses a one-step RT-PCR combined with Taqman technology to detect the novel coronavirus’s ORF1ab gene and N gene. If both the ORF1ab gene and the N gene are positive (Ct < 38), the test result is determined to be positive.

### Statistical analysis

Continuous variables were expressed as a median and interquartile range, and categorical variables were expressed as counts and percentages. Mann–Whitney U test was used for comparison between the two groups for continuous variables, the Kruskal-Wallis test for multiple comparisons, and *χ*^2^ and Fisher test for categorical variables. All statistical analyses were performed using IBM SPSS 22.0 statistical software, and graphs were plotted using GraphPad 9.0. *p* ≤ 0.05 was defined as a statistically significant difference.

## Results

### Characteristics of the study participants

A total of 157 cases of Omicron variants infection and 144 cases of prototype strain infection were included in this study. Table 1 summarizes the clinical characteristics of the subjects, including gender, age, BMI, symptoms, and underlying diseases. There were 81 males (51.6%) and 76 females (48.4%) in the omicron group, 77 males (53.5%) and 76 females (46.5%) in the prototype strain group. The median age of patients infected with the omicron variant is younger than that of the prototype strain (35.0 vs. 47.0, *p* < 0.001). Patients infected with the omicron variant had a lower median BMI than the prototype strain (22.5 vs. 24.4, *p* = 0.015). Hypertension (6.3%) and diabetes mellitus (1.9%) were the most common underlying diseases for omicron variant infections, and most infected patients had no specific underlying diseases.

These results indicate a general population susceptibility to Omicron variants; about 50% of patients with Omicron variants infection had first symptoms of cough, fever, sore throat, and ground glass/splatter showed by the CT image; compared with patients with prototype strain infection, the proportion of patients with lung solid lesions was lower. Laboratory parameters showed higher lymphocyte counts and eGFR levels in Omicron variants-infected patients compared to prototype strain-infected patients, which also indicated that Omicron variants caused less damage to the immune system and renal function of infected patients. The laboratory test results are detailed in the Supplementary material.

Notably, the median time from onset to hospitalization (2 vs. 6) days and the median length of hospitalization (15 vs. 22) days of Omicron variants infected patients were shorter than those of the prototype strain.

### Comparison of the dynamic changes in Ct values of Omicron and prototype strain genes in different sample types

**Figure 2:**
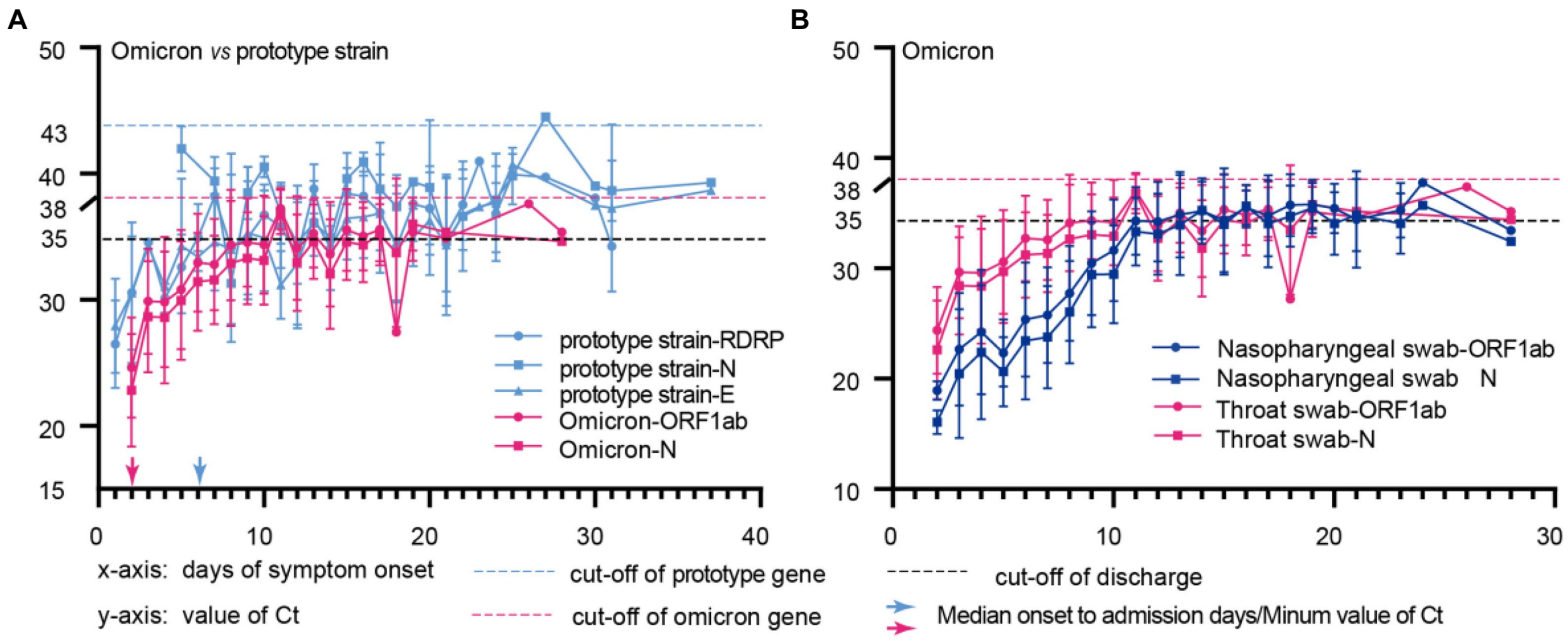
Comparison of the dynamic changes in Ct values of Omicron and prototype strain genes in different sample types. **(A)**, Throat swab from patients with omicron or prototype strain infection; **(B)**, Throat swabs and Nasopharyngeal swabs of Omicron infected patients; The x-axis represents the time of disease course since symptom onset, and the y-axis shows the Ct values for each feature of prototype strain(blue) and Omicron(red) in different sample types. The blue and red arrows represent the median days from onset to admission for omicron and prototype strain-infected patients.

We compared the changes of Ct values longitudinally among patients after Omicron variants and prototype strain infections, and the data showed that patients had the lowest Ct values at the point of admission (highest viral nucleic acid load) (Figure 2A); Omicron strain infected patients had the lowest Ct values at a median time of 2 (2–3) days from the onset to admission, while prototype strain group had the lowest Ct values at a median time of 6 (4–10) days from the onset to hospital admission (Figure 2A); the duration of Omicron variant infection was 15 (13–18) days, while that of patients infected with the prototype strain was 22 (13–28) days (Table 1). The above results suggest that the viral load of Omicron variants peaks 2–3 days after the onset, and the time for the test result to turn negative in Omicron variants infection is 6–10 days shorter than that in prototype strain infection. Further study found that the Omicron variant infection group had lower nucleic acid Ct values in nasopharyngeal swabs than in throat swabs, and the Ct values reached the same at 12 days.

We compared Ct values between patients’ nasopharyngeal and throat swabs at admission. The Ct values of nasopharyngeal swabs were lower than those of throat swabs (ORF1ab gene 21.72 vs. 29.67, *p* < 0.001; N gene 19.5 vs. 28.59, *p* < 0.001), and the latters fluctuated during the recovery period (Figure 2B). The above results suggest that the viral load of nasopharyngeal swabs is higher and stable in the early stage of viral infection. Therefore, nasopharyngeal swabs should be the primary samples for early detection. And Omicron variants should be diagnosed and quarantined as soon as symptoms appear to cut off the source of infection in the shortest possible time.

### Comparison of Ct dynamic changes in Omicron-infected patients with symptoms or no symptoms

**Figure 3:**
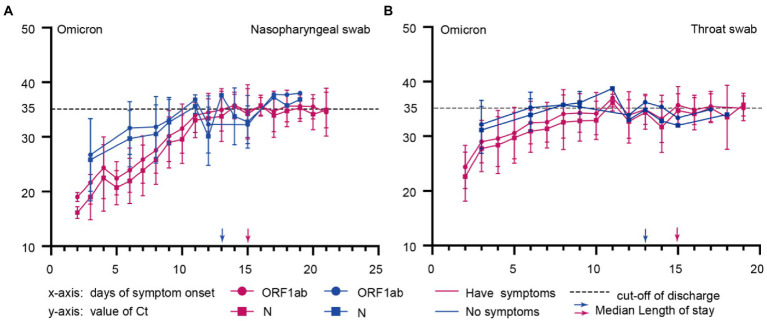
Comparison of Ct dynamic changes in omicron-infected patients with symptoms or no symptoms. **(A)**, Nasopharyngeal swabs of Omicron infected patients with symptoms or no symptoms; **(B)**, Throat swabs of Omicron infected patients with symptoms or no symptoms; The x-axis represents the time of disease course since symptom onset, and the y-axis shows the Ct values for Omicron infected patients with symptoms or without symptoms. Blue and red represent asymptomatic and symptomatic infected persons, respectively.

**Figure 4:**
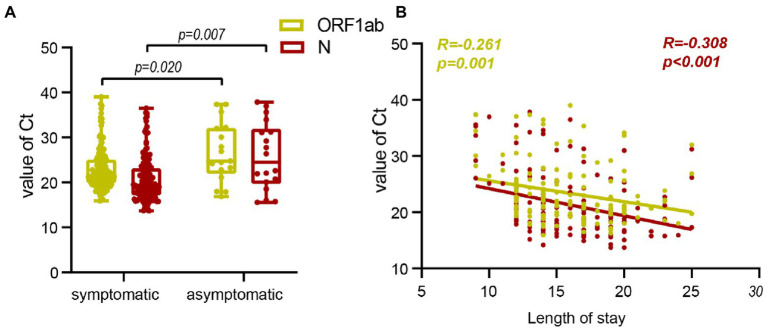
Correlation between Ct value and symptoms onset and length of stay. **(A)**, Comparison of admission Ct values between patients with symptoms and asymptomatic patients. **(B)**, Correlation between admission Ct value and length of stay.

Approximately 90% of patients with domestic epidemic Omicron variant infections in China are asymptomatic (Zhang et al., 2022). We further investigated the dynamics of Ct values in symptomatic and asymptomatic individuals. In the Omicron variant cohort, 75 subjects provided the vaccination information. 2 (2.7%) patients were not vaccinated against the COVID-19 vaccine, 2(2.7%) patients were vaccinated with one dose, 27 (36.0%) patients were vaccinated with two doses, and 44 (58.7%) patients received a booster vaccination. The patient has no record of reinfection. We found that symptomatic ones with lower Ct values at admission early in the infection (ORF1ab gene, *p* = 0.02; N gene, *p* = 0.007; Figures 3A, 4A) had a longer length of stay than asymptomatic ones (15 vs. 13) days (*p* = 0.006; Figure 3; Table 1).

### Correlation between Ct value and symptoms onset and length of stay

Correlation analysis was conducted to further assess the difference between symptomatic and asymptomatic clinical indicators of Ct after Omicron variants infection. It showed a negative correlation between lower admission Ct values and longer length of stay (Figures 3, 4B).

## Discussion

We systematically described the clinical characteristics of 157 Omicron variants-infected patients and the dynamic changes of Omicron variants virus loads and disease progression in 1,723 multi-type samples. The dynamic changes of the viral loads after prototype strain infection were also compared. Our study found that the median time of peak viral replication in respiratory samples from Omicron variant-infected patients was 2 days after onset and the lowest point of nucleic acid Ct values. The viral load decreased as the disease progressed, with a viral duration of 15 (13–18) days in Omicron variants. And prototype strain infection, the peak time of viral replication was 6–10 days after the onset, which is consistent with a previous study by Zheng et al. (Zheng et al., 2020; Salvatore et al., 2021), the duration of viral shedding was 22 (13–28) days. Boucau et al. (2022) reported that the viral load started to decay from the first PCR positive or symptom onset. However, the duration of viral shedding in the Omicron variant in this study failed to be characterized as well.

Since Omicron variants replicate better in the upper respiratory tract than previous variants, it has been proposed that saliva or oral specimens may be more sensitive than nasopharyngeal samples in detecting Omicron (Marais et al., 2021; University of Hong Kong, 2021). Our study found that in the early stages of Omicron variants infection, the nucleic acid Ct value of nasopharyngeal swabs was lower than that of throat swabs. Both of them reached the same Ct value in 12–15 days of the disease, while Ct values of throat swabs were more likely to fluctuate during the recovery period, which may lead to false negatives in nucleic acid detection. This is detrimental to the prevention and control of the epidemic because of sampling errors and instability of throat swabs. Ct values of Nasopharyngeal swabs have been stable during the recovery period, and therefore nasopharyngeal swabs still have high analytical sensitivity and stability for RT-PCR (Matic et al., 2022). However, oral specimens are still an option, although attention should be paid to the lower viral load, especially when there may be difficulties in obtaining nasopharyngeal swabs (Williams et al., 2020).

The asymptomatic carrier rate of Omicron variants is much higher than that of other variants. In the outbreak of Omicron variants in Shanghai, 90.8% of infected patients were asymptomatic (Zhang et al., 2022). This high rate of asymptomatic infection may be a significant factor in this variant’s widespread and rapid global spread (Garrett et al., 2022; Zhang et al., 2022). The mean Ct value at 30.1 in asymptomatic Omicron patients, higher than that in symptomatic ones at 25.9, increased with the number of days of symptoms (Pollock et al., 2021; Schrom et al., 2022). We studied the admission Ct value of asymptomatic individuals and found that the admission Ct value was negatively correlated with the length of stay. This will help to strengthen the management of asymptomatic infection patients and shorten the time for nucleic acid transformation.

Several limitations should be considered when interpreting this study’s results. First, we only selected the subjects infected with the Omicron variant in one city as the study population. However, there may be differences between cities and regions. In addition, the sample size for our analysis is not large enough, which may affect the statistical power. Therefore, further generalization and external validity studies are needed to clarify the intrinsic mechanism.

## Conclusion

In conclusion, the viral load of Omicron variants reached its peak value 2–3 days after the onset and the duration of virus was 15–18 days; The viral load of symptomatic patients was higher, and the overall length of hospitalization was longer, and the two indicators were negatively correlated; In the early stage of Omicron variants infection, nasopharyngeal swab test is still the first choice and shows better stability in the later stage. Supplementary material showed higher lymphocyte counts and eGFR levels in those infected with the Omicron variant compared to those infected with the prototype strain, suggesting that the Omicron variant causes less damage to the immune system and renal system function in those infected. This study provides a basis for the development of prevention and control strategies. Once symptoms appear, diagnosis and quarantine should be carried out as soon as possible, the source of infection should be cut off in the shortest time, and closed-loop management measures.

## Data availability statement

The original contributions presented in the study are included in the article/Supplementary material, further inquiries can be directed to the corresponding authors.

## Ethics statement

This study was approved by the Ethics Committee of Taizhou Hospital, Zhejiang Province (K20210218). Informed consent was obtained from each subject. Minors’ participation in the study was approved by their parents and/or legal guardians.

## Author contributions

HZ and BS supervised the project. BS, KZ, and T-HT designed the study. KZ organized the data and wrote the manuscript. KZ, BH, XZ, HC, JP, YZ, XB, MC, and JXu collected clinical data and provided clinical supervision. All authors contributed to the article and approved the submitted version.

## Funding

This work is supported by the Zhejiang Provincial Medical and Health Science and Technology Program (2019RC089), Zhejiang Provincial Natural Science Foundation of China (Grant No. LTGY23H290007), Sichuan Provincial Natural Science Foundation of China (Grant No. 23NSFSC1861), and Scientific Research Fund of Zhejiang Provincial Education Department (Y202146073).

## Conflict of interest

The authors declare that the research was conducted in the absence of any commercial or financial relationships that could be construed as a potential conflict of interest.

## Publisher’s note

All claims expressed in this article are solely those of the authors and do not necessarily represent those of their affiliated organizations, or those of the publisher, the editors and the reviewers. Any product that may be evaluated in this article, or claim that may be made by its manufacturer, is not guaranteed or endorsed by the publisher.

## Supplementary material

The Supplementary material for this article can be found online at: https://www.frontiersin.org/articles/10.3389/fmicb.2022.1037733/full#supplementary-material
