# Supplementary material for: Longitudinal observation of viral load in patients infected with Omicron variant and its relationship with clinical symptoms
Source: Front Microbiol. 2023 Jan 13;13:1037733. doi: 10.3389/fmicb.2022.1037733 (PMC9880150; doi:10.3389/fmicb.2022.1037733)
Supplement: Supplementary file 1 [file Data_Sheet_1.docx]

## Supplementary figure legends

**Supplementary figure 1. Comparison of laboratory parameters between omicron and prototype strain infected subjects.** Differences in organ function between Omicron and prototype strain infected subjects, including liver function, renal function, lung function, cardiac function, blood electrolytes, complete blood count, and coagulation.

**Supplementary figure 1 Comparison of laboratory parameters between omicron and prototype strain infected subjects**
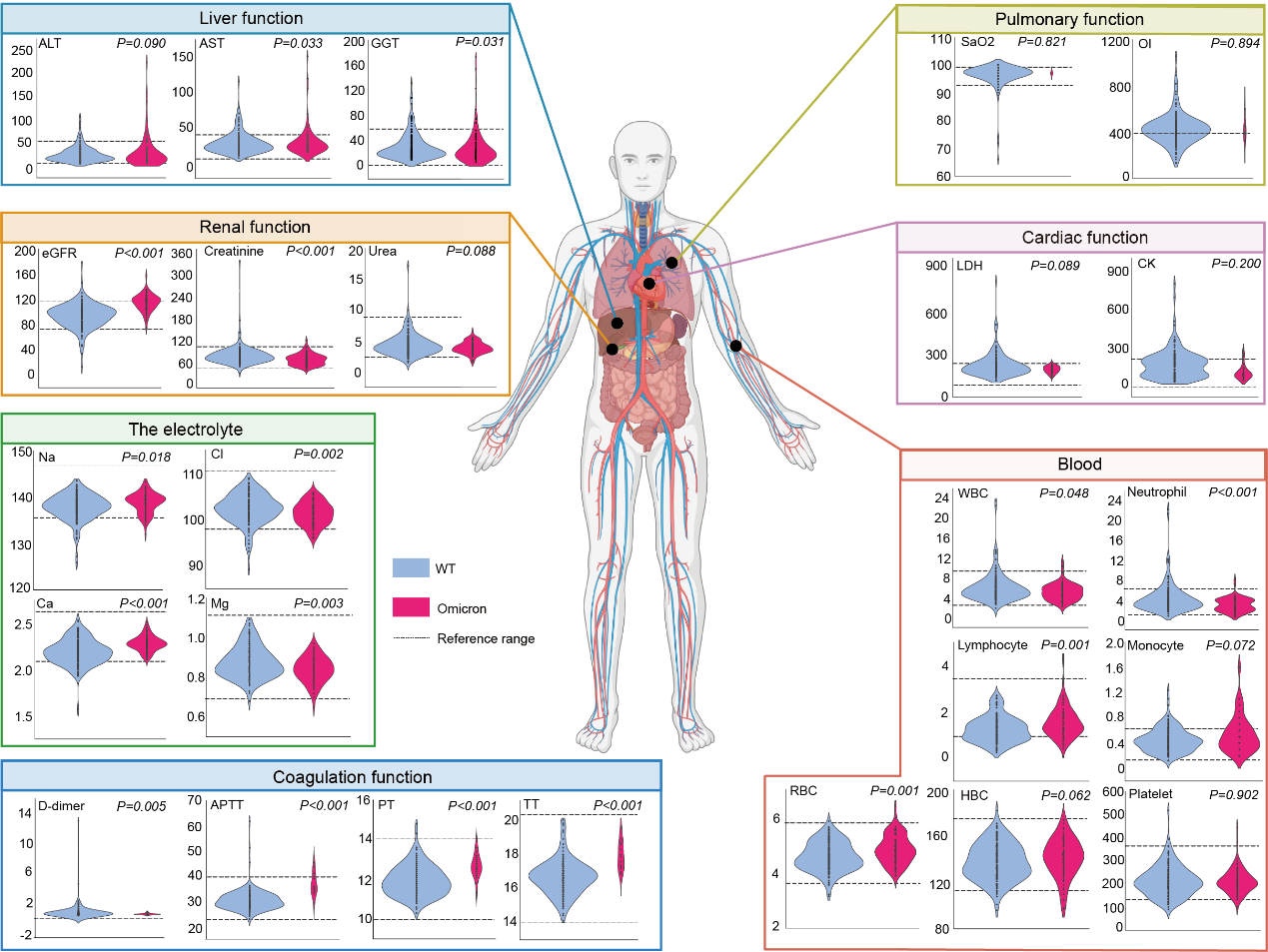


**Supplementary Table 1. Comparison of laboratory parameters between omicron and prototype strain infected subjects**

| [**Laboratory**](javascript:;) [**index**](javascript:;)  **Median(IQR)** | **Omicron**  **(n=157)** | **Prototype strain**  **(n=144)** | ***P value*** |
| --- | --- | --- | --- |
| [**Complete**](javascript:;) [**blood**](javascript:;) [**count**](javascript:;) | | | |
| WBC | 4.95(3.90-6.10) | 5.24(4.31-6.77) | **0.048** |
| Neutrophil | 2.80(1.90-3.90) | 3.40(2.60-5.09) | **<0.001** |
| Lymphocyte | 1.40(1.13-1.90) | 1.18(0.80-1.58) | **0.001** |
| Monocyte | 0.50(0.30-0.68) | 0.42(0.31-0.54) | 0.072 |
| RBC | 4.77(4.45-5.17) | 4.50(4.18-4.96) | **0.001** |
| Hb | 143(130-154) | 135(126-151) | 0.062 |
| Platelet | 204(181-239) | 204(165-248) | 0.902 |
| **Coagulation function** |  |  |  |
| D-dimer | 0.22(0.16-0.26) | 0.24(0.18-0.41) | **0.005** |
| **Liver function** | |  |  |
| ALT | 16.5(11.0-32.5) | 20.9(14.0-34.2) | 0.090 |
| AST | 21.0(17.0-28.8) | 24.0(19.0-30.0) | **0.033** |
| GGT | 20.5(14.0-34.8) | 23.5(17.0-37.3) | **0.031** |
| **Renal function** |  |  |  |
| eGFR | 117.0(106.0-122.0) | 95.0(83.0-105.0) | **<0.001** |
| Creatinine | 67.0(54.0-76.8) | 74.0(66.0-88.0) | **<0.001** |
| Urea | 3.83(3.30-4.45) | 4.01(3.25-5.21) | 0.088 |
| **Pulmonary function** |  |  |  |
| SaO2 | 97.0(96.5-97.5) | 97.0(96.0-98.0) | 0.821 |
| [Oxygenation](javascript:;) [index](javascript:;) | 405.0(357.0-526.0) | 420.7(348.3-485.7) | 0.894 |
| **Cardiac function** |  |  |  |
| LDH | 192.0(172.0-211.0) | 200.0(170.0-240.3) | 0.089 |
| CK | 78.0(58.5-108.5) | 68.5(44.0-89.3) | 0.200 |
